# Superior prognosis stratification for stage III colon cancer using log odds of positive lymph nodes (LODDS) compared to TNM stage classification: the Japanese study group for postoperative follow-up of colorectal cancer

**DOI:** 10.18632/oncotarget.27692

**Published:** 2020-08-18

**Authors:** Shimpei Ogawa, Michio Itabashi, Yoshiko Bamba, Masakazu Yamamoto, Kenichi Sugihara

**Affiliations:** ^1^Department of Surgery, Institute of Gastroenterology, Tokyo Women’s Medical University School of Medicine, Tokyo, Japan; ^2^Department of Surgery, Tokyo Medical and Dental University, Tokyo, Japan

**Keywords:** colon cancer, LODDS, TNM, staging, prognosis

## Abstract

Objectives: The aim of this study is to examine whether prognosis stratification in staging of Stage III colon cancer using T factor and log odds of positive lymph nodes (LODDS) categories is superior to that of the TNM staging system.

Materials and Methods: The subjects were 5,919 patients with Stage III colon cancer who underwent curative resection at 24 Japanese institutions. Univariate analysis of LODDS categories and clinicopathologic factors was conducted using a Cox proportional hazards regression model for cancer-specific survival (CSS). Independent prognostic factors for CSS were extracted in multivariate analysis using factors with significance in univariate analysis. Effect sizes of risk factors for CSS were compared using the LogWorth statistic. Combinations of T factor and LODDS categories were used to create L-stage subgroups A, B and C. Stratification of prognosis with L-stage and TNM was compared using the Akaike information criterion (AIC).

Results: In multivariate analysis, LODDS was identified as an independent prognostic factor, together with age, maximum tumor diameter, histopathological grade, L, V, pT, and pN. The LogWorth of LODDS was 17.149, which was the second highest after pT (31.562), and that of pN was 7.434. The 5-year CSS was 96.5%, 88.5%, and 66.6% in TNM stages IIIA, IIIB, and IIIC, respectively, and 96.0%, 87.6%, and 59.3% in L-stage A, B, and C, respectively (*p* < 0.0001). AICs for TNM and L-stage were 14,795.5 and 14,707.8, respectively.

Conclusions: Prognosis stratification of the stage classification for Stage III colon cancer was superior with L-stage compared to TNM stage classification.

## INTRODUCTION

Staging of colon cancer is widely used as an indicator for selection of therapeutic strategy and prognostic prediction. The TNM staging system of the American Joint Committee on Cancer (AJCC) and the Union International Cancer Control (UICC) is the most widely used worldwide [1]. Lymph node metastasis (LNM) is an important prognostic factor in this system [2, 3]. The N grade in TNM staging is determined based on the number of LNMs, but does not reflect the number of lymph node dissections (LNDs). However, this number may also be a prognostic factor in colon cancer, with more LNDs suggesting a more favorable prognosis [4–6]. More LNDs also reduces stage migration and enables correct grading [7, 8]. Therefore, the number of LNDs may have a large impact on grading of the N category in TMN staging.

The lymph node ratio (LNR) and the log odds of positive lymph nodes (LODDS) are used to reflect the numbers of LNDs and LNMs in staging [9, 10]. LODDS is calculated based on the numbers of lymph nodes that are positive and negative for metastasis, and has been reported to be useful for selection of high-risk patients, prognosis prediction, and stratification for patients with breast, gastric, and colorectal cancers [2, 11–15]. In TNM staging, Stage III colon cancer is stratified into subgroups: Stage IIIA, Stage IIIB, and Stage IIIC in T and N categories. A more precise staging system may be possible using LODDS, in which the number of LNDs is added instead of the N category, but this has not been examined to date. Therefore, the aim of this study is to examine whether a staging system for Stage III colon cancer based on the T category and LODDS enables stratification of patients to give a homogeneous and more accurate prognosis, compared to TNM staging.

## RESULTS

### LODDS category

The mean numbers of LNDs and LNMs in the subjects were 24.5 ± 14.8 and 2.89 ± 3.13, respectively. Three cut-off points were extracted based on classification and regression trees (CART) analysis (-1.711, -0.717, 0.077). LODDS was classified into four categories: A ≤ -1.711; B > -1.711 to ≤ -0.717; C > -0.717 to ≤ 0.077; and D > 0.077, which included 3648, 1604, 459 and 208 patients, respectively (Table 1).

**Table 1:** Clinicopathologic characteristics of patients

Characteristic	Value
Age (median; range)	67 (19–99)
Gender	
Male	3163 (53.4)
Female	2756 (46.6)
Maximum tumor diameter (median; range)	41.0 (11–135)
Histopathological grade	
G1, G2	5385 (91.0)
G3, G4	534 (9.0)
Lymphatic invasion	
L0	1268 (21.4)
L1	4651 (78.6)
Venous invasion	
V0	1526 (25.8)
V1	4393 (74.2)
Postoperative adjuvant therapy	
No	2246 (37.9)
Yes	3673 (62.1)
Number of retrieved lymph nodes	
< 12	966 (16.3)
≥ 12	4953 (83.7)
Pathologic *T* stage	
T1	285 (4.8)
T2	510 (8.6)
T3	3456 (58.4)
T4a	1307 (22.1)
T4b	361 (6.1)
Pathologic *N* stage	
N1a	2437 (41.2)
N1b	2002 (33.8)
N2a	1005 (17.0)
N2b	475 (8.0)
LODDS category	
LODDS A	3648 (61.6)
LODDS B	1604 (27.1)
LODDS C	459 (7.8)
LODDS D	208 (3.5)

### Risk factors

**Figure 1:**
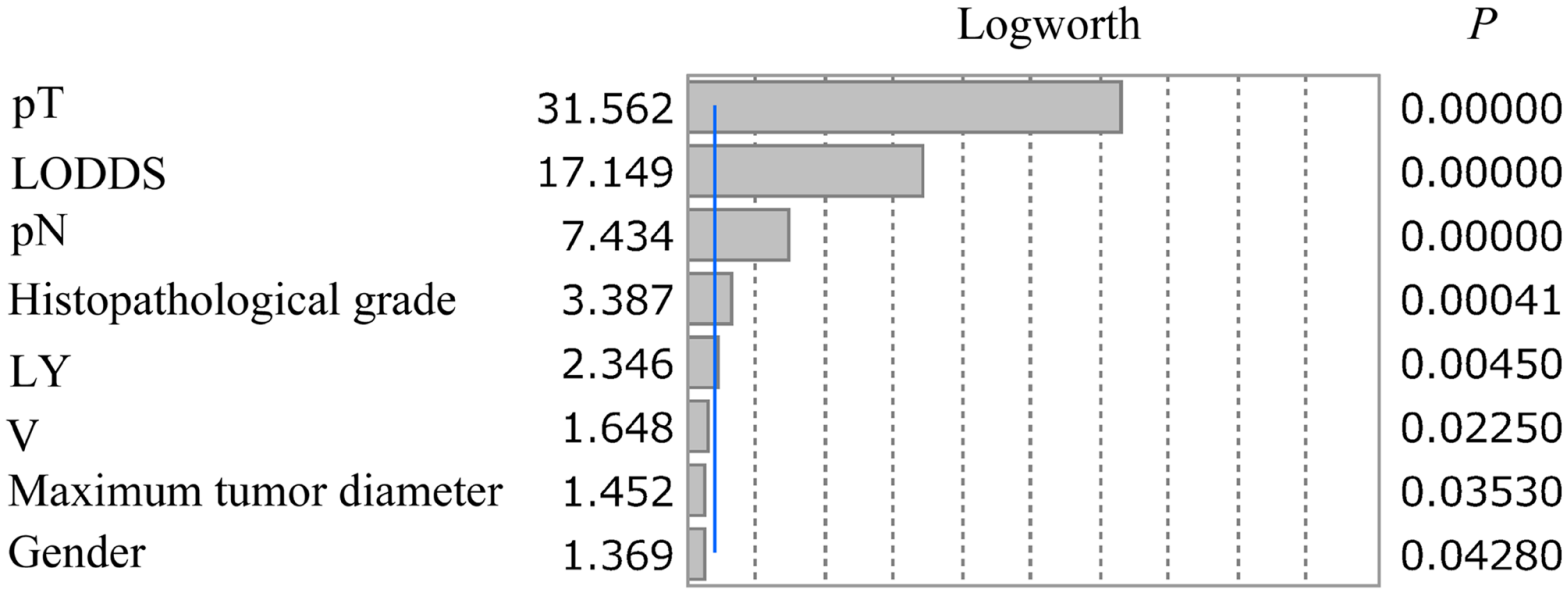
LogWorth of independent risk factors identified in multivariate analysis. The LogWorth of pT was 31.562, followed by 17.149 for LODDS category and 7.434 for pN. The blue line indicates a LogWorth of 2.

Among the 5,919 subjects, 908 (15.3%) had cancer-specific deaths. Cox proportional hazards regression analysis was used to identify factors associated with cancer-specific survival (CSS). In univariate analysis, gender, maximum tumor diameter, histopathological grade, lymphatic invasion (L), venous invasion (V), pathologic T stage (pT), pathologic N stage (pN) and LODDS category were significantly related to CSS. In multivariate analysis, male gender, maximum tumor diameter (≥ 41.0 mm), histopathological grade (G3 + G4), L1, V1, pT (T3, T4a, T4b), pN (N1b, N2a, N2b) and LODDS (B, C, D) were independent factors associated with CSS (Table 2). The LogWorth for pT of 31.562 was highest, followed by 17.149 for LODDS category and 7.434 of pN, suggesting that the LODDS category was more important than pN (Figure 1).

**Table 2:** Univariate and multivariate analysis of risk factors for cancer-specific survival

Factor	Number of patients (%)	Univariate analysis			Multivariate analysis		
		Hazard ratio	95% CI	*P*	Hazard ratio	95% CI	*P*
Age							
< 68	3012 (50.9)	1					
≥ 68	2907 (49.1)	1.13	0.99–1.29	0.0682			
Gender							
Male	3163 (53.4)	1.14	1.00–1.30	0.0499	1.15	1.00–1.31	0.0423
Female	2756 (46.6)	1			1		
Maximum tumor diameter							
< 41.0	2947 (49.8)	1			1		
≥ 41.0	2972 (50.2)	1.49	1.31–1.70	< 0.0001	1.16	1.01–1.33	0.0347
Histopathological grade							
G1, G2	5385 (91.0)	1			1		
G3, G4	534 (9.0)	1.93	1.59–2.31	< 0.0001	1.41	1.16–1.70	0.0007
Lymphatic invasion							
L0	1268 (21.4)	1			1		
L1	4651 (78.6)	1.90	1.58–2.31	< 0.0001	1.33	1.10–1.62	0.0034
Venous invasion							
V0	1526 (25.8)	1			1		
V1	4393 (74.2)	1.68	1.42–2.00	< 0.0001	1.22	1.03–1.46	0.0200
Postoperative adjuvant therapy							
No	2246 (37.9)	1.07	0.93–1.22	0.3296			
Yes	3673 (62.1)	1					
Number of retrieved lymph nodes							
< 12	966 (16.3)	1					
≥ 12	4953 (83.7)	1.04	0.87–1.26	0.6485			
Pathologic T stage							
T1	285 (4.8)	1			1		
T2	510 (8.6)	2.06	0.98–4.32	0.0418	1.79	0.85–3.75	0.1066
T3	3456 (58.4)	4.52	2.34–8.75	< 0.0001	3.32	1.70–6.46	< 0.0001
T4a	1307 (22.1)	9.57	4.93–18.57	< 0.0001	5.94	3.03–11.63	< 0.0001
T4b	361 (6.1)	14.54	7.38–28.67	< 0.0001	9.09	4.55–18.16	< 0.0001
Pathologic N stage							
N1a	2437 (41.2)	1			1		
N1b	2002 (33.8)	1.65	1.39–1.96	< 0.0001	1.37	1.14–1.64	0.0008
N2a	1005 (17.0)	2.44	2.02–2.94	< 0.0001	1.56	1.25–1.95	< 0.0001
N2b	475 (8.0)	5.57	4.57–6.77	< 0.0001	2.29	1.76–2.99	< 0.0001
LODDS							
LODDS A	3648 (61.6)	1			1		
LODDS B	1604 (27.1)	1.70	1,46–1.97	< 0.0001	1.29	1.08–1.54	0.0054
LODDS C	459 (7.8)	2.69	2.18–3.30	< 0.0001	1.69	1.31–2.17	< 0.0001
LODDS D	208 (3.5)	6.99	5.59–8.67	< 0.0001	3.57	2.70–4.71	< 0.0001

Abbreviations, CI: confidence interval.

### L-staging

**Figure 2:**
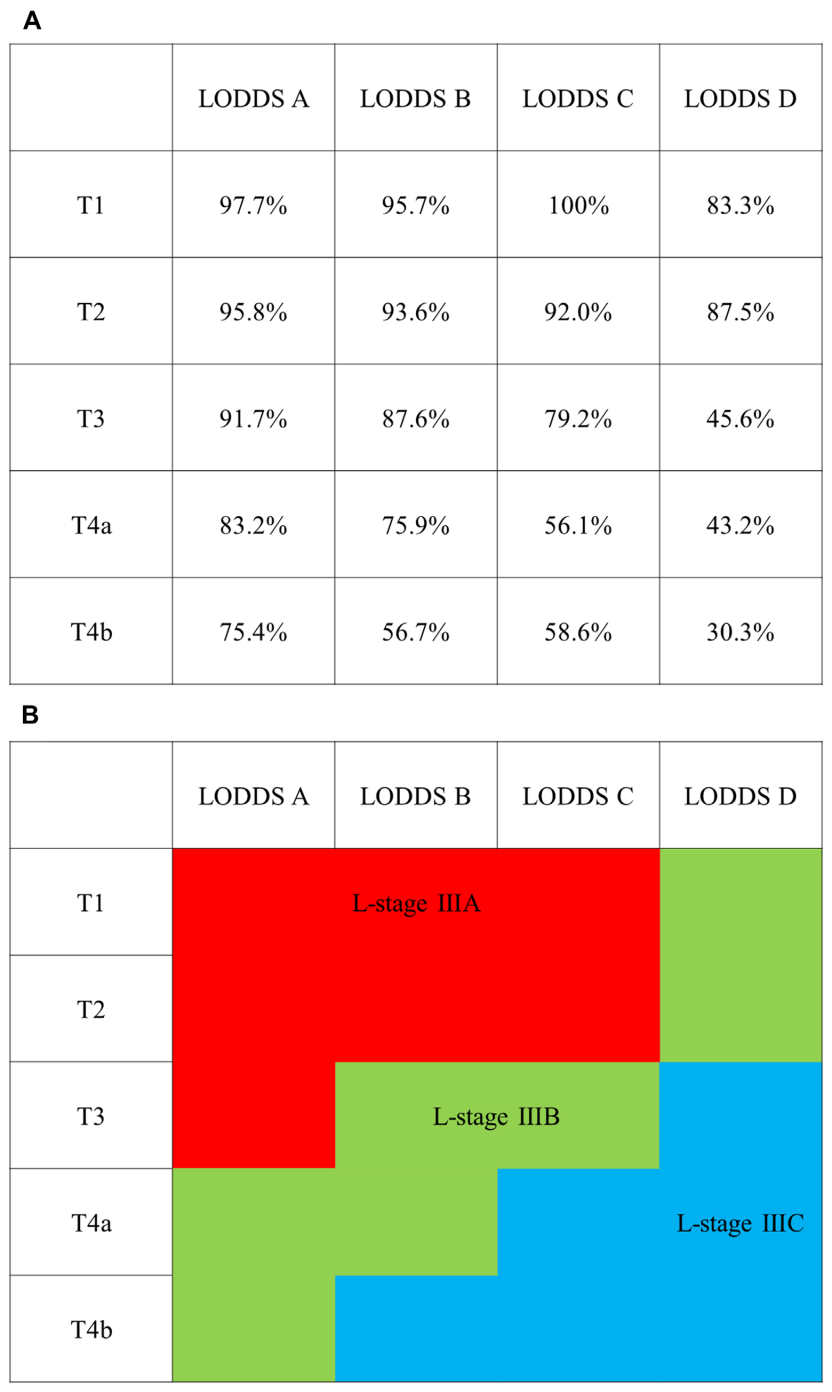
Classification of L-stage subgroups based on 5-year cancer specific survival (CSS). (**A**) 5-year CSS in pathologic T and LODDS category. (**B**) L-stage IIIA: 5-year CSS ≥ 90% (red), L-stage IIIB: 5-year CSS ≥ 70% to < 90% (green), and L-stage IIIC: 5-year CSS < 70% (blue).

Twenty combinations of pT and LODDS category were used to establish three subgroups (L-stage A, B and C) based on CSS. Combinations of T1 and LODDS A, B, and C; T2 and LODDS A, B, and C; and T3 and LODDS A, which had CSS ≥ 90%, were included in L-stage A. Combinations of T1 and LODDS D; T2 and LODDS D; T3 and LODDS B and C; T4a and LODDS A and B; and T4b and LODDS A, which had CSS ≥ 70% to < 90%, were included in L-stage B. Other combinations were included in L-stage C (Figure 2A and 2B).

**Figure 3:**
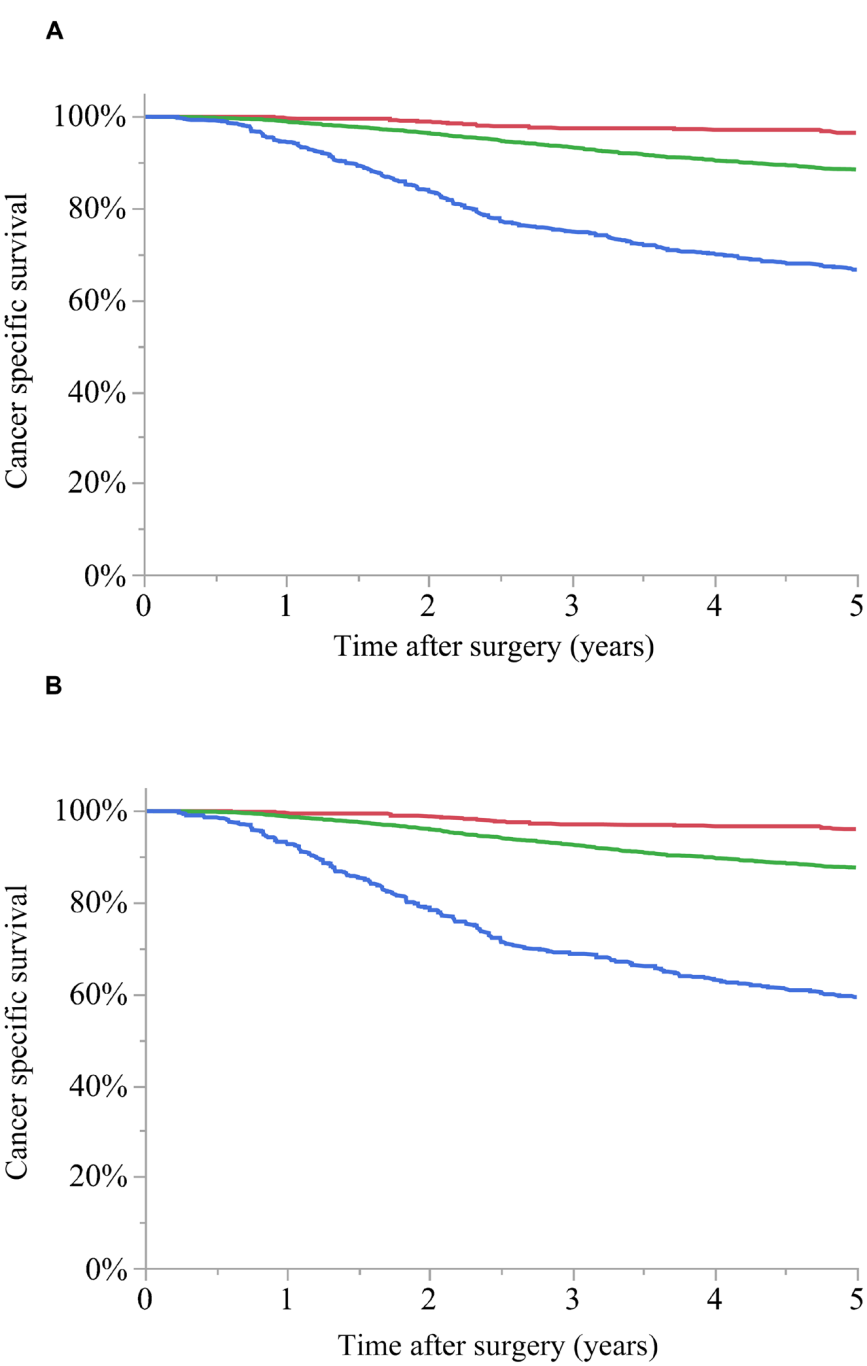
Cancer-specific survival (CSS) curves for stage III colon cancer. (**A**) TNM stage: 5-year CSS for stage IIIA (red): 96.5%, stage IIIB (green): 88.5%, and stage IIIC (blue): 66.6% (*p* < 0.0001). (**B**) L-stage: 5-year CSS for A (red): 96.0%, B (green): 87.6%, and C (blue): 59.3% (*p* < 0.0001). The Akaike information criterion value was 14,795.5 for TNM stage and 14,707.8 for L-stage.

The 5-year CSS was 96.5%, 88.5%, and 66.6% in TNM stages IIIA, IIIB, and IIIC, respectively (*p* < 0.0001), and 96.0%, 87.6%, and 59.3% in L-stages A, B, and C, respectively (*p* < 0.0001). The Akaike information criterion (AIC) values for TNM stage and L-stage were 14,795.5 and 14,707.8, respectively, with a lower value for L-stage (Figure 3A and 3B).

## DISCUSSION

The lower AIC value for L-staging for Stage III colon cancer, based on pT and LODDS categories, compared to that for TNM staging, suggests that stratification using L-staging is superior to that with TNM staging. This may be due to the impact of the LODDS category. In multivariate analysis, pT, pN, and LODDS category were independently associated with CSS, in addition to gender, maximum tumor diameter, histopathological grade, L, and V. The highest LogWorth of 31.562 was found for pT, followed by 17.149 for LODDS category and 7.434 for pN. Logworth is calculated as -log_10_ (*p* value), and higher values are considered to be more significant [16]. In this study, pT, LODDS, and pN were extracted as independent risk factors for CSS in multivariate analysis, with *P* < 0.0001 for each factor. Thus, the individual impact of each risk factor for CSS could not be compared based on the *P* value, but LogWorth values could be used for this comparison. Thus, the higher LogWorth for the LODDS category compared to the N category suggests the importance of the LODDS category in stratification of CSS. Thus, cases with a poor prognosis can be selected using the LODDS category. The hazard ratio (HR) of N2b vs. N1a in pN was 2.29, whereas HR for LODDS D vs. A was 3.57, which also indicates that cases with a poor prognosis are better identified using the LODDS category.

The number of LNDs in patients with colon cancer depends on surgical technique, quality of histopathological examination, tumor biology, and patient-tumor immunologic response [17–20]. More LNDs has been associated with a more favorable prognosis in LNM-positive and LNM-negative cases [4–6]. This may be because high-quality surgery results in appropriate and probably increased LNDs. In addition, micrometastases and isolated tumor cells, which are difficult to identify in routine pathological examinations, may be removed by LND [7, 21]. A high-quality histopathological examinations allows accurate evaluation of the N category, and this reduces the number of underestimated and missed cases for which adjuvant therapy is required, which is also likely to improve the therapeutic outcome. These findings show that the number of LNDs is an important factor that should be taken into consideration in stratification and prediction of outcome in N staging. Since LODDS is calculated based on the numbers of negative and positive LNMs and LNDs, staging is reflected more accurately.

LNR is also used as an indicator that includes the number of LNDs in N staging [9, 10]. LNR is calculated by dividing LNMs by LNDs, and is a useful prognostic factor in colon cancer. Ceelen et al. [10] found that LNR was an independent prognostic factor for Stage III colon cancer in a meta-analyses of 16 studies. However, there are problems with use of LNR. For example, if all LNDs are positive for metastasis, LNR is equal to 1, regardless of the total number of LNDs, and all cases with LNR of 1 are classified in the same category. However, prognoses can differ based on the total numbers of LNMs and LNDs.

In contrast, in LODDS, 0.5 is added to the number of LNMs and to the number of lymph nodes negative for metastasis, and thus the value differs depending on the number of LNMs, even when the numbers of LNMs and LNDs are the same. For example, LODDS is 1.099 for a case with 1 LND and 1 LNM, but 2.708 for a case with 7 LNDs and 7 LNMs. In addition, since the difference becomes larger in cases with a smaller number of LNMs (that is, cases with fewer LNDs), stratification can be performed for these cases. In fact, cases with < 12 LNDs may not be appropriately stratified by LNR, but with LODDS such cases can be stratified [9, 22–24]. In addition, in LNM-negative Stage I or II cases, LNR is 0 regardless of the number of LNDs, but with LODDS, the value differs depending on the number of LNDs [2, 25]. These properties show the utility of LODDS as a prognostic factor and in N staging.

The current study suggests that L-staging can be performed using LODDS. The AIC value was lower than that for TNM staging, and thus L-staging may better stratify the prognosis of patients with Stage III colon cancer. The 5-year CSS was almost the same in TNM stage IIIA and L-stage A, and in TNM stage IIIB and L-stage B, but differed between TNM stage IIIC (66.6%) and L-stage C (59.3%). This suggests that cases with a poor prognosis may be better identified using the L-staging system. Postoperative adjuvant therapy for Stage III colon cancer is not uniform, and various drugs and administration periods are used. To select this therapy, accurate stratification of stage is required, and the L-staging system enables more accurate stratification, compared to TNM staging. This suggests that L-staging could contribute to planning of optimum regimens of drugs and administration periods for individual patients.

This study has some limitations. It was a retrospective study of cases in high-volume centers in Japan, and further cases are needed for prospective analysis. The chosen cutoff value in the LODDS category classification has a large impact on stratification by L-staging. Cutoffs were calculated using CART, but more appropriate cutoffs may further improve the L-staging system. It is also unclear if this system can be used for Stage I or II cases, including those with preoperative treatment, rectal cancer, many dissected lymph nodes, and a LNM-negative status, which were excluded from this study.

## MATERIALS AND METHODS

### Patients

The subjects were 5,919 patients with Stage III colon cancer (excluding appendiceal cancer) who underwent curative resection between January 1997 and December 2012 at 24 Japanese institutions, all of which were in the Japanese Study Group for Postoperative Follow-up of Colorectal Cancer (JFUP-CRC). No patients received neoadjuvant therapy. The inclusion criteria were a pathological diagnosis of colon cancer, complete clinicopathological factors (age, gender, histopathological grade, maximum tumor diameter, L, V, pT, pN, number of lymph nodes analyzed, number of positive lymph nodes, postoperative adjuvant treatment), and time and vital status at the last follow-up clearly noted. Stages are reported using the TNM classification in the UICC Staging Manual. This study was approved by the Central Institutional Review board (Tokyo Medical and Dental University) and local ethical committee.

### LODDS

LODDS values are defined as log ([pLN + 0.5]/[nLN + 0.5]), where pLN and nLN are the numbers of positive and negative lymph nodes, respectively. A value of 0.5 is added to the numerator and denominator to avoid a singularity error. To investigate optimal categorization of LODDS, CART was used to determine highly discriminating cutoffs for CSS, using the R software package ver. 3.5.3 (The R Foundation for Statistical Computing Platform: x86_64-w64-mingw32/x64). CSS was defined as time from surgery to death due to cancer recurrence. Based on three obtained cutoffs, LODDS was divided into four categories (A, B, C, D).

### Risk factors

Univariate analysis of LODDS category and other clinicopathologic factors was conducted using a Cox proportional hazards regression model for CSS. Independent prognostic factors for CSS were extracted based in multivariate analysis using factors with significance in univariate analysis. Effect sizes of independent factors on CSS were compared using LogWorth (where LogWorth is -log10 [*p*-value], such that *p* = 0.01 is equivalent to a LogWorth of 2.0, *p* = 0.001 is denoted by a LogWorth of 3.0, etc.) for further interpretative clarity [16].

### Construction of the L-staging system

Twenty combinations of T category (T1, T2, T3, T4a, T4b) and LODDS category (A, B, C, D) were divided into 3 subgroups based on CSS to construct a L-staging system.

### Statistical analysis

Survival curves were generated using the Kaplan–Meier method and compared by log-rank test. Comparison of stratification of survival outcomes using TNM stage and L-stage was performed using the AIC calculated in a Cox proportional hazards regression model to identify the better system for predicting outcomes. A lower AIC value was considered to be optimal. In each analysis, *P* < 0.05 was taken to be significant. All analyses were performed using JMP Pro ver.14 for Windows^®^ (SAS Institute Inc., Cary, NC, USA).

## CONCLUSIONS

Our results suggest that a staging system using LODDS for Stage III colon cancer may stratify prognosis more accurately than the TNM staging system. This is important because accurate stratification of prognosis will enable individual adjustment of treatments appropriate for the estimated prognosis, which should lead to a better therapeutic outcome.

## Abbreviations

AJCC: American Joint Committee on Cancer
UICC: Union for International Cancer Control
LNM: lymph node metastasis
LND: lymph node dissection
LNR: lymph node ratio
LODDS: log odds of positive lymph nodes
CART: classification and regression trees
CSS: cancer-specific survival
L: lymphatic invasion
V: venous invasion
pT: pathologic T stage
pN: pathologic N stage
AIC: Akaike information criterion
HR: hazard ratio
JFUP-CRC: Japanese Study Group for Postoperative Follow-up of Colorectal Cancer
